# Behavioral Profiles in Romanian Resident Physicians: An Exploratory Study with Implications for Medical Education and Human Resource Development

**DOI:** 10.3390/healthcare14172844

**Published:** 2026-09-03

**Authors:** Dragoș Nicolae Nicolescu, Ana Cernega, Simona Pârvu, Vlad-Gabriel Vasilescu, Lucian-Toma Ciocan, Marina Imre, Ovidiu Popa-Velea, Iuliana Raluca Gheorghe, Silviu-Mirel Pițuru

**Affiliations:** 1 Department of Organization, Professional Legislation and Management of the Dental Office, Faculty of Dental Medicine, “Carol Davila” University of Medicine and Pharmacy, 17-23 Plevnei Street, 020021 Bucharest, Romania; dragos-nicolae.nicolescu@drd.umfcd.ro (D.N.N.); silviu.pituru@umfcd.ro (S.-M.P.); 2 National Institute of Public Health, Faculty of Medicine, “Carol Davila” University of Medicine and Pharmacy, 050474 Bucharest, Romania; simona.parvu@umfcd.ro; 3 Discipline of Dental Prosthesis Technology, Faculty of Dentistry, “Carol Davila” University of Medicine and Pharmacy, Dionisie Lupu Street, No. 37, District 2, 020021 Bucharest, Romania; lucian.ciocan@umfcd.ro; 4 Discipline of Prosthodontics, Faculty of Dentistry, “Carol Davila” University of Medicine and Pharmacy, 37 Dionisie Lupu Street, District 2, 020021 Bucharest, Romania; marina.imre@umfcd.ro; 5 Department of Medical Psychology, Faculty of Medicine, “Carol Davila” University of Medicine and Pharmacy, 050474 Bucharest, Romania; ovidiu.popa-velea@umfcd.ro; 6 Department of Marketing and Medical Technology, Faculty of Medicine, “Carol Davila” University of Medicine and Pharmacy, 050474 Bucharest, Romania; raluca.gheorghe@umfcd.ro

**Keywords:** medical education, human resource development, behavioral assessment, resident physicians, interpersonal competencies, emotional regulation

## Abstract

**Background/Objectives**: Early residency is not only about building clinical knowledge. It also involves learning how to communicate, collaborate, and regulate one’s behavior throughout real-world pressure—skills that shape teamwork and everyday patient encounters. Describing the behavioral profiles of early-career physicians can therefore be useful for mapping strengths and developmental needs in training. **Methods**: A cross-sectional exploratory study was conducted between May and June 2023 among 83 medical residents from a major Romanian training centre. Participants completed the Harrison Assessment, which yields trait-level scores relevant to workplace functioning. Individual traits were summarized, and complementary characteristics were examined as trait pairs, using a balance/imbalance classification to describe configuration-level profiles. **Results**: Across five of the six trait pairs, non-balanced configurations represented 60.24–65.06% of the cohort; Delegation showed a larger balanced group (55.42%). These distributions describe heterogeneity within the participating cohort at one time point and do not establish developmental sequence, competence, or clinical consequence. **Conclusions**: Configuration-level profiling may offer a descriptive way to examine how complementary behavioral tendencies co-occur. Replication, population-specific psychometric evaluation, longitudinal follow-up, and validation against external outcomes are required before educational or clinical utility can be inferred.

## 1. Introduction

The development of medical professionals is shaped not only by clinical knowledge and procedural skills, but also by behavioral and interpersonal competencies that influence performance in real healthcare settings. Previous research emphasizes that professional success in the medical field does not depend solely on professional competencies; it is also influenced by other factors, namely transversal competencies—communication abilities, emotional regulation, quality of teamwork, effective decision-making, and reflective capacity [1,2,3,4]. These competencies contribute to how individuals adapt to clinical environments and manage the interpersonal demands inherent in the doctor–patient relationship, ultimately, facilitating the successful implementation of the treatment plan and improving patient health outcomes.

In a broader context, professional development is understood as a dynamic process in which individuals evolve not only in terms of measurable performance, but also with respect to their needs, aspirations, and internal motivations [5,6]. Similarly, classical learning theories describe the acquisition of competence as a staged evolution from lack of awareness to autonomous mastery [7,8,9], highlighting the critical role of experience and self-reflection in medical training.

In clinical contexts, these developmental processes rely heavily on behavioral tendencies—patterns that influence how individuals communicate, collaborate, assume responsibility, tolerate uncertainty, and respond to interpersonal or emotional demands. In this sense, pre-existing behavioral vulnerabilities may be amplified by the high cognitive load and fluctuating emotional demands characteristic of early-career medical professionals. This can also have long-term effects, especially in shaping adaptation to clinical environments [10,11,12,13]. Emotional intelligence has been repeatedly linked to these processes, supporting self-awareness, empathy, and effective social functioning, which are essential for constructive doctor–patient communication and teamwork [14,15,16,17]. Throughout this manuscript, behavioral tendencies denote self-reported preferences for particular ways of acting in work-related situations. They are distinguished from personality traits, which describe relatively stable, decontextualized dispositions; from competencies, which are criterion-referenced capabilities demonstrated against an external performance standard; and from interpersonal competencies, the subset of competencies enacted in interaction with patients and colleagues. The present study measures behavioral tendencies only and does not assess competence.

Moreover, developmental and learning theories describe early professional training as a stage of ongoing consolidation, in which competencies are not yet predictably expressed across contexts and are still shaped through experience and reflective practice [18,19]. In clinical training environments, consolidation may be uneven across domains, and cognitive and procedural development may progress earlier relative to socio-emotional consolidation, generating configuration-level asymmetries relevant to professional functioning [20,21]. In parallel, the organizational and medical education literature suggests that demographic factors may influence interpersonal functioning and communication-related tendencies, supporting the exploration of gender-related differences in behavioral configurations [22,23]. Taken together, these studies support examining behavioral tendencies as configurations rather than as isolated attributes, because complementary tendencies may be represented unevenly within the same individual. Emotional intelligence is considered part of the broader conceptual context through which behavioral preferences may relate to interpersonal functioning; it was not measured in this study. The cross-sectional analyses do not test a sequence of professional development.

Despite the growing interest in the role of behavioral and emotional factors in medical education, many studies tend to examine these elements separately. Although communication skills, emotional intelligence, reflective ability, and decision-making tendencies have been examined individually in many research papers, few studies have investigated how these components coexist within the same individual, or how imbalances across behavioral domains may create vulnerabilities relevant to clinical training. Moreover, empirical evidence on the distribution of such behavioral configurations among medical residents remains relatively limited, despite residency representing a formative stage characterized by rapid development, increasing autonomy, and significant interpersonal exposure [24]. Three limitations of this literature are relevant here. First, most studies are variable-centered, estimating associations between attributes and outcomes across individuals rather than describing how attributes co-occur within them. Second, the attributes most relevant to practice have generally been examined one at a time, so the question of whether a strength in one domain coexists with, compensates for, or masks a limitation in a complementary domain has rarely been posed. Third, person-centered work in medicine has more often addressed students and specialty preference than physicians already in postgraduate training. The present study addresses these limitations by applying a person-centered, configuration-level analysis of complementary trait pairs in a resident cohort. Describing behavioral tendencies in resident physicians may help identify questions for future research on professional functioning and education [25,26]. The present study does not measure performance, competence, developmental need, or the effect of an educational intervention; any such application requires independent outcome-based validation.

The prior literature suggests that interpreting traits in isolation may be insufficient, as practice-relevant behaviors often emerge from the co-occurrence and interaction of complementary (or competing) tendencies. Also, configuration-level approaches can identify meaningful profiles (i.e., recurring patterns of traits within individuals) that are not captured by single-trait interpretations [27,28]. Accordingly, the objectives of this exploratory study were: (1) to describe the distribution of behavioral tendencies among medical residents at trait level; (2) to characterize these tendencies at configuration level, using six predefined pairs of complementary tendencies; (3) to explore whether these configurations differed by gender; and (4) to examine whether recurring configuration profiles could be identified across the cohort. No educational method or intervention was evaluated in this study; the educational implications set out in Section 4.2 are proposed on the basis of the descriptive findings and were not themselves tested.

## 2. Materials and Methods

*Study Design*: This study used a cross-sectional exploratory design, in order to examine behavioral tendencies among medical residents, including those that are associated with potential vulnerabilities. Behavioral instruments frequently employed in medical education and Organizational Psychology were used in order to analyze interpersonal functioning, decision-making preferences, and adaptive capacities in early-career professionals [1,3,4]. The study design aligns with previous research exploring non-technical skill development and dispositional factors influencing clinical performance. The study is reported in accordance with the STROBE statement for cross-sectional studies.

*Participants and Recruitment*: Participants were recruited from residency programs in General Medicine and Dentistry at “Carol Davila” University of Medicine and Pharmacy in Bucharest, Romania. One hundred residents received invitations to participate, with 83 of them completing the study instruments. Inclusion criteria were active enrollment during the data collection period (May–June 2023) and provision of informed consent; exclusion criteria included incomplete questionnaires or withdrawal. Participation was voluntary and therefore self-selected. Residents who chose to complete a behavioral assessment may differ from those who did not, for example, in openness to feedback, self-awareness, or available time. As no information was collected from non-participants, the direction and magnitude of this potential selection bias cannot be estimated; the distributions are accordingly interpreted as descriptive of the participating cohort rather than representative of the eligible population.

*Ethical Considerations*: The study received approval from the Scientific Research Ethics Committee of “Carol Davila” University of Medicine and Pharmacy (Approval No. 13048/17.05.2022) and was conducted in accordance with the Declaration of Helsinki. Participation was voluntary, and all responses were anonymized to ensure confidentiality.

*Instrument*: There are many personality assessment instruments used in Psychology, Education, Healthcare, and Organizational Psychology. Some examples include the Big Five Personality Traits [29], the Myers-Briggs Type Indicator (MBTI) [30], the HEXACO model of personality structure [31], and the Minnesota Multiphasic Personality Inventory (MMPI) [32]. These instruments are widely used to assess personality traits, preferences, emotional tendencies, and psychological characteristics in both clinical and non-clinical settings [33]. However, many researchers argue that personality instruments have important limitations in capturing actual behaviors in real-world situations [34]. One major limitation is that the personality instruments are often descriptive, but not predictive of the behavior. For example, the Big Five model measures broad traits such as openness, conscientiousness, extraversion, agreeableness, and neuroticism. However, these traits do not always predict how a person will behave under pressure, in social conflict, or across changing environments, thus being strongly influenced by context [35]. Another limitation is the issue of contextual variability, meaning that many personality instruments are supported by the assumption that traits remain relatively stable across situations, but behavioral research shows that individuals may behave differently depending on stress, culture, workplace expectations, or social norms [36,37].

In this study, behavioral tendencies were assessed with the Harrison Assessment, which reports work-related preference scores on a 2–10 scale [38,39,40,41,42]. Within the instrument’s scoring convention, 6 is the arithmetic midpoint and is described as the neutrality threshold; the investigators did not derive or optimize this value from the present data. Six predefined pairs of complementary tendencies were examined jointly. A pair was classified as balanced when both scores were at least 6; the remaining configurations comprised two directionally distinct partial-balance categories and one double-low category. For the gender comparisons and latent class analysis these three categories were aggregated as non-balanced because several individual quadrant cells were sparse. The four-category distributions are nevertheless reported in Section 3.2, together with the continuous score summaries. This dichotomisation necessarily discards within-category variation and treats observations close to the threshold as categorically different; alternative thresholds were not examined.

The Harrison Assessment was selected because its focus on preferences expressed in work settings corresponds to the construct examined here and, more specifically, because its predefined complementary pairs allow each tendency to be interpreted in relation to its counterpart. General personality frameworks such as the Big Five or HEXACO address broader dispositional structure and would not provide the same paired architecture without constructing pairings post hoc. A licensed Romanian-language edition was available, and the platform provided participants with an individual report. These practical and conceptual considerations explain the selection; they do not establish superior validity.

The published psychometric literature available for the Harrison Assessment is less extensive and less independent than the literature supporting established general personality frameworks. The supporting evidence cited here [41,42] is derived mainly from developer or provider documentation, and no physician- or resident-specific validation study was identified. The analytical dataset available for this study contained calibrated trait scores rather than item-level responses; consequently, Cronbach’s α, McDonald’s ω, construct validity, and measurement invariance could not be re-estimated in this cohort. The instrument is therefore used as a structured exploratory profiling tool, not as a population-validated measure of competence or clinical performance. Accordingly, throughout this manuscript the Harrison Assessment is used solely as an exploratory measure of self-reported, work-related behavioral tendencies, and its psychometric properties and its applicability to resident-physician populations remain to be independently established.

The licensed Romanian-language edition was administered through the provider’s online platform. The external documentation cited in references [41,42] reports test–retest reliability and associations with job-performance criteria; these estimates were not independently re-estimated in the present sample and should not be interpreted as physician-population validation.

*Procedure*: Participants accessed the online assessment through personalized secured links. Upon completion, they received an individualized, automatically generated report. The anonymized dataset used for analysis included trait scores and associated behavioral dimensions. Behavioral assessments of this type are frequently used in research as a tool potentially supporting self-awareness and professional development among healthcare trainees [10,11].

*Statistical Analysis*: Data analysis was conducted using IBM SPSS Statistics 25. Quantitative distributions were assessed with the Shapiro–Wilk test, and descriptive statistics were reported as means with standard deviations or medians with interquartile ranges, as appropriate. Categorical variables were summarized as counts and percentages. Latent class analysis (LCA) was performed in R 4.4.0 with poLCA using eight binary indicators: the balance status of the six predefined trait pairs and the two standalone dimensions, Organization and Warmth/empathy. Candidate solutions were compared using log-likelihood, AIC, BIC, entropy, class size, and interpretability; the reported two-class solution had maximum log-likelihood −403.5756, AIC 841.1513, BIC 882.2715, and entropy 0.793. Given n = 83, eight indicators, and a smaller modal class of 15 participants, the solution is treated as a sample-specific, exploratory description. No resampling, split-sample validation, or independent replication was performed, and model stability cannot be inferred from fit indices alone. Gender comparisons used Fisher’s exact tests. Six comparisons were conducted without multiplicity correction; *p* values are therefore nominal, and any single result is interpreted as hypothesis-generating. No missing data were observed for the analyzed variables, so analyses used the full sample (*n* = 83).

## 3. Results

The study included 83 medical residents (80.7% female; 19.3% male). Participant characteristics are summarized in Table 1. Regarding their educational background, 54.2% were graduates of the Faculty of Dental Medicine, while 45.8% had completed their studies at the Faculty of General Medicine.

The main distinction between these two categories lies in the professional licensing pathway: graduates of the Faculty of Dental Medicine obtain the right to independent practice immediately upon completion of their 6-year degree, whereas graduates of the Faculty of General Medicine are required to complete a medical residency program specific to their chosen specialty in order to gain both competence and the right to practice independently.

### 3.1. Analysis of Individual Traits

Table 2 provides a comprehensive overview of the traits identified within the investigated target group. It includes the description of each analyzed trait, the mean and median scores, as well as the score ranges for each trait. Additionally, the table indicates the percentage of study participants who exhibited an innate preference for a specific trait, defined by a score above 9.

Descriptive analysis showed that, at the group level, the medians for most traits were centered around a score of 6. According to the Harrison Assessment interpretation guidelines, this score corresponds to the neutrality threshold. At the same time, the observed score ranges indicate meaningful inter-individual variability within the cohort (Table 2).

Only a small proportion of participants scored in the strong innate preference zone (above 9) for any given trait. For example, 10.84% of respondents displayed a strong innate predisposition toward self-acceptance or assertiveness, 8.43% toward helpfulness, and 10.84% toward diplomacy or frank/direct communication.

Several traits showed comparatively higher representation in the strong innate zone. Nearly half of the residents (44.58%) obtained scores above 9 on self-improvement, indicating a pronounced tendency toward setting ambitious goals and seeking continuous development. Organization (28.91%) and warmth/empathy (30.12%) also had higher proportions of strong innate scores relative to other traits, suggesting that structured task management and expressed emotional connection are reasonably well-represented, although not dominant across the group.

At the level of aggregated tendencies above the neutrality threshold (score ≥ 6), the mean number of such traits per respondent was 4.05 ± 1.34, with a median of 4 (interquartile range 3–5) and a range of 2–8. Most trait scores were moderate, with stronger expression confined to a subset of traits. Because no hypotheses were formally specified and all measures were obtained once, this result is reported descriptively and does not establish a developmental process.

### 3.2. Trait Pair Analysis

Beyond the individual trait analysis, a central objective of this study was to examine how traits interact in trait pairs, because these combinations provide a more specific picture of behavioral patterns relevant to medical practice [20,21]. In this regard, twelve of the previously described traits are grouped into six trait pairs, while Warmth/empathy and Organization are analyzed separately, as they are not integrated into any trait pair:

- Diplomatic and Frank/direct—***Communication*** trait pair;
- Risk analyzer and optimistic—***Strategic thinking*** trait pair;
- Self-acceptance and Self-improvement—***Self-approach*** trait pair;
- Assertive and Helpful—***Relationship control*** trait pair;
- Open/reflective and Self-confident—***Approach to opinions*** trait pair;
- Assumed authority and Collaborative—***Delegation*** trait pair.

**Figure 1:**
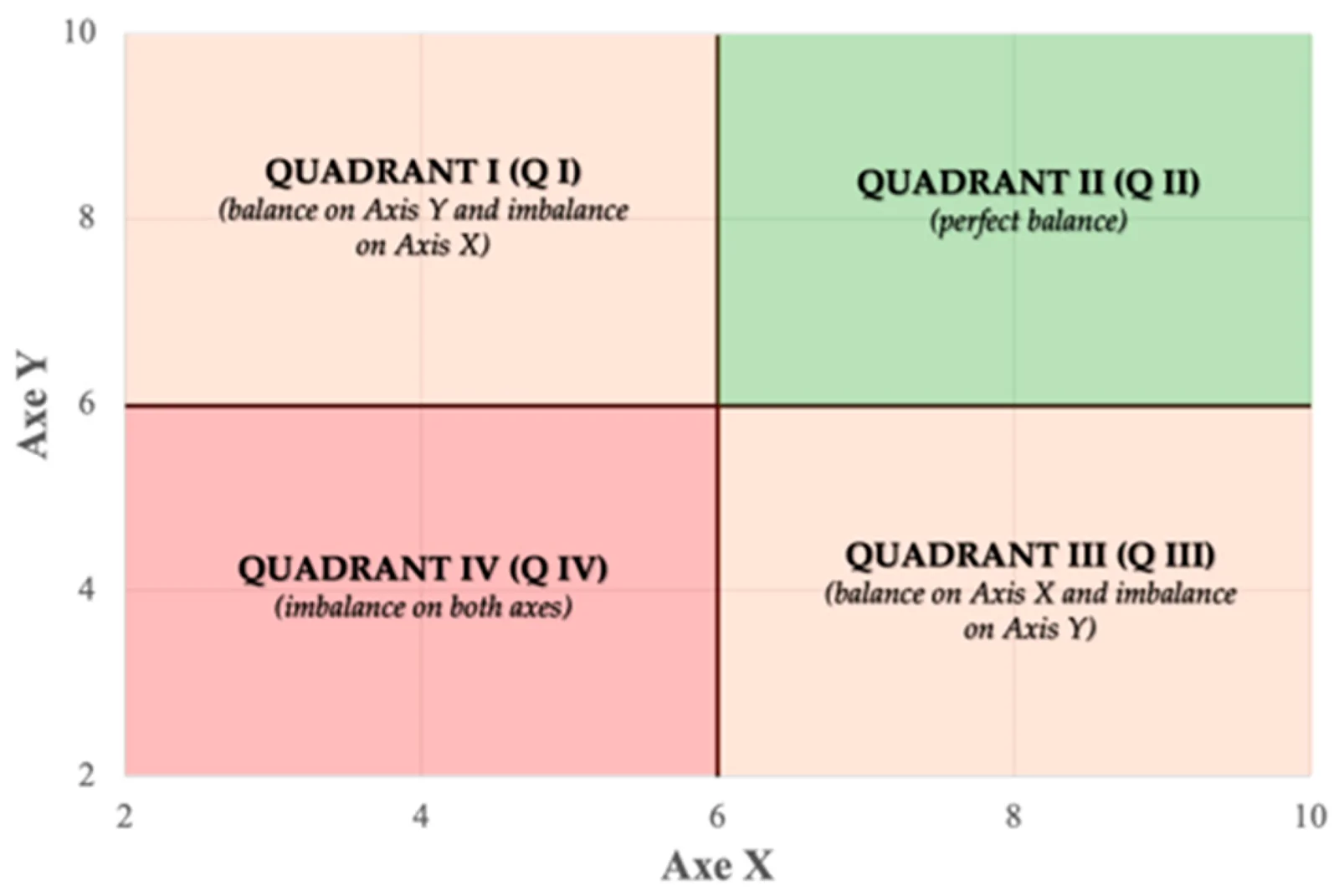
Example of a graphical representation of the trait pair.

In the following section, we will provide a descriptive analysis of each trait pair. As shown in Figure 1, the trait pairs are based on the connection between two axes, X and Y, each corresponding to one of the traits that forms the foundation of the trait pair.

For each trait pair, scores on the two traits were plotted on orthogonal axes. A score of 6 was used as the neutrality threshold on both axes, dividing the graph into four quadrants: Quadrant II (both traits ≥ 6) corresponding to functional balance, Quadrants I and III representing partial balance (one trait ≥ 6 and the other < 6), and Quadrant IV indicating imbalance on both dimensions (both traits < 6). This quadrant-based representation served as a descriptive tool for visualizing how residents were distributed across balanced and imbalanced configurations within each trait pair.

Given the focus of the present study, results are reported in terms of balance versus imbalance at the trait pair level, as well as the distribution of residents across the four quadrants for trait pair.

Quadrant labels used throughout this section (e.g., “diplomacy-dominant”, “risk-analysis-dominant”) are descriptive summaries of trait patterns, not clinical categories. Each is defined operationally at first use, naming the tendency that reached the neutrality threshold and the complementary tendency that did not, and each is given in quotation marks to signal its descriptive status. They are not validated clinical diagnoses, confirmed risk categories, or measured behavioral deficits. No external criterion measure, such as a performance outcome, wellbeing indicator, supervisor rating, or clinical incident record, was collected in this study, and no configuration reported below has been linked to any clinical outcome. Behavioral interpretations of these configurations are advanced in Section 4.1.2, where they are presented as hypotheses for future criterion-validation research rather than as findings of the present study.

#### 3.2.1. The Communication Trait Pair (Diplomatic—Frank/Direct)

At the core of effective communication lies the individual’s ability to adjust their message, according to the context and the audience. This requires finding a balance between the tendency to react in a straightforward manner and the need to introduce a degree of diplomatic subtlety when the situation demands it.

Thus, the Communication trait pair examines the balance between the tendency to communicate frankly and directly and the opposite tendency, to use diplomacy in interpersonal interactions. The distribution of residents by balance status and gender is presented in Table 3.

Among the respondents, 34.94% showed a balanced profile in Communication, while 65.06% presented a non-balanced configuration. The proportion of balanced profiles was similar in females (35.8%) and males (31.2%), with no statistically significant difference between them (Fisher’s exact test, *p* = 1.000).

**Figure 2:**
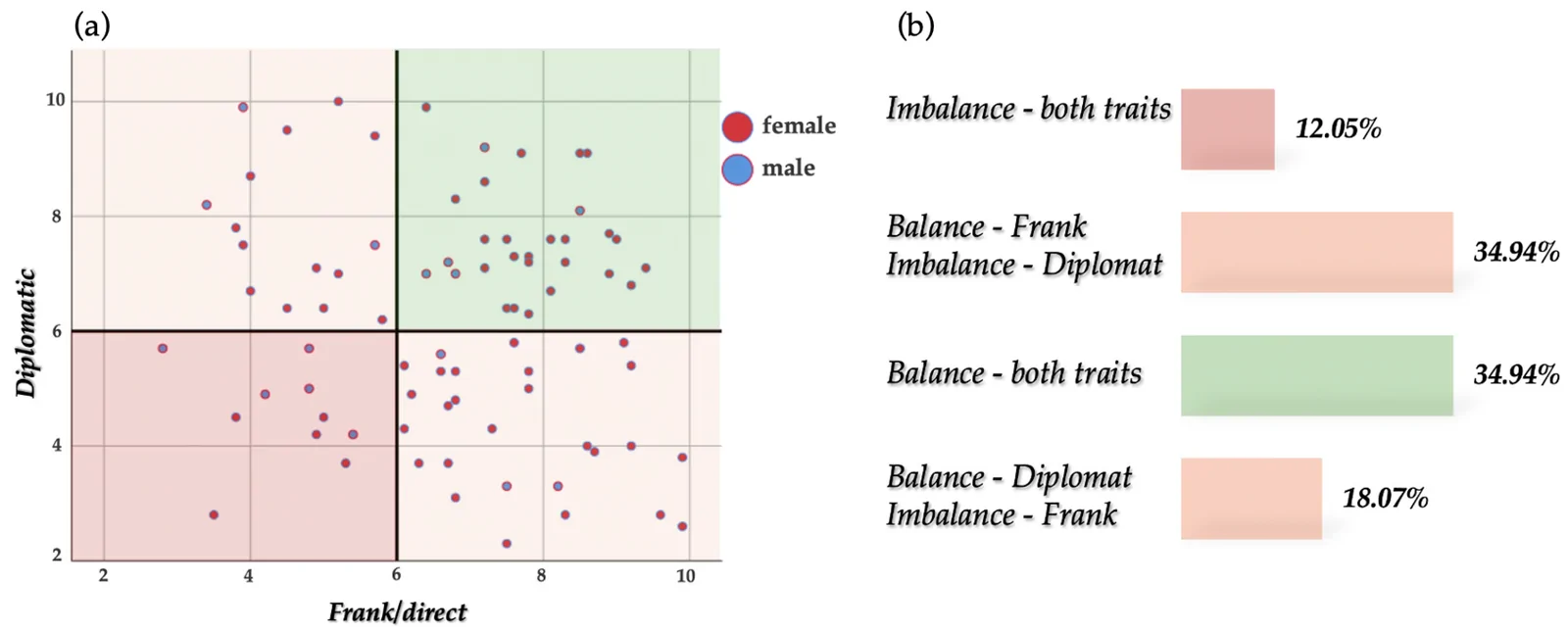
(**a**) Distribution of participants within the Communication trait pair (Frank/direct vs. Diplomatic), by gender and balance quadrants. (**b**) Distribution of participants within the Communication trait pair (Frank/direct vs. Diplomatic), by balance quadrants.

The distribution of residents across the quadrants of the Communication trait pair is illustrated in Figure 2a,b. Overall, 18.07% of participants were located in Quadrant I, corresponding to a diplomacy-dominant configuration (Diplomatic ≥ 6, Frank/direct < 6). 34.94% of residents fell within Quadrant III, reflecting a directness-dominant configuration (Frank/direct ≥ 6, Diplomatic < 6). 12.05% were classified in Quadrant IV, showing a configuration in which neither tendency reached the neutrality threshold.

Among the non-balanced Communication configurations, directional asymmetry between the two tendencies was more common than a configuration in which both scores were below the neutrality threshold. This is a descriptive distribution and does not establish functional impairment or a developmental process.

#### 3.2.2. The Strategic Thinking Trait Pair (Risk Analyzer–Optimistic)

The Strategic thinking trait pair analyzes the balance between the tendency to analyze risks and the tendency toward optimism. Table 4 displays the distribution of residents by gender and balance status.

In this dimension, 38.55% of residents presented a balanced profile, while 61.45% showed a non-balanced configuration. The proportion of balanced profiles was 35.8% among females and 50.0% among males, with no statistically significant difference (*p* = 0.393).

**Figure 3:**
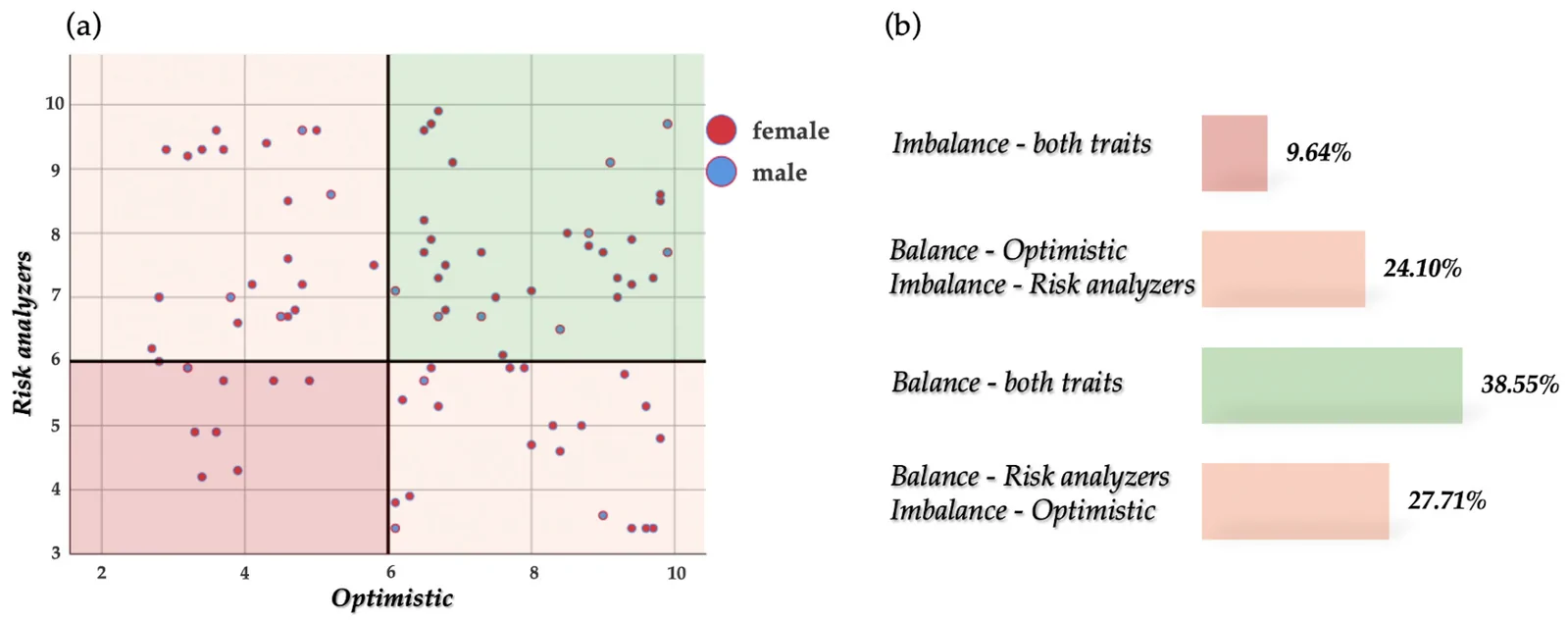
(**a**) Distribution of participants within the Strategic thinking trait pair (Risk analyzers vs. Optimistic) by gender and balance quadrants. (**b**) Distribution of participants within the Strategic thinking trait pair (Risk analyzers vs. Optimistic) by balance quadrants.

As shown in Figure 3a,b, 27.71% (*n* = 23) of residents were positioned in Quadrant I, corresponding to a risk-analysis-dominant configuration (Risk analyzer ≥ 6, Optimistic < 6). 24.10% (*n* = 20) were classified in Quadrant III, reflecting an optimism-dominant configuration (Optimistic ≥ 6, Risk analyzer < 6). 9.64% (*n* = 8) of participants fell within Quadrant IV, indicating that neither tendency reached the neutrality threshold. Finally, 38.55% (*n* = 32) were positioned in Quadrant II, representing a balanced profile.

These findings suggest that, although a substantial subgroup combined risk analysis and optimism in a balanced manner, most non-balanced profiles reflected asymmetry between the two complementary tendencies rather than simultaneously low levels in both traits.

#### 3.2.3. The Self-Approach Trait Pair (Self-Acceptance–Self-Improvement)

The Self-approach trait pair examines the interaction between Self-acceptance and Self-improvement. At group level, 39.76% of residents were located in the balanced area, while 60.24% showed a non-balanced configuration. Gender distributions and balance status are shown in Table 5.

Among female residents, 38.8% had a balanced profile and 61.2% an imbalanced one; among males, 43.8% were balanced and 56.3% imbalanced, with no statistically significant difference (*p* = 0.780).

**Figure 4:**
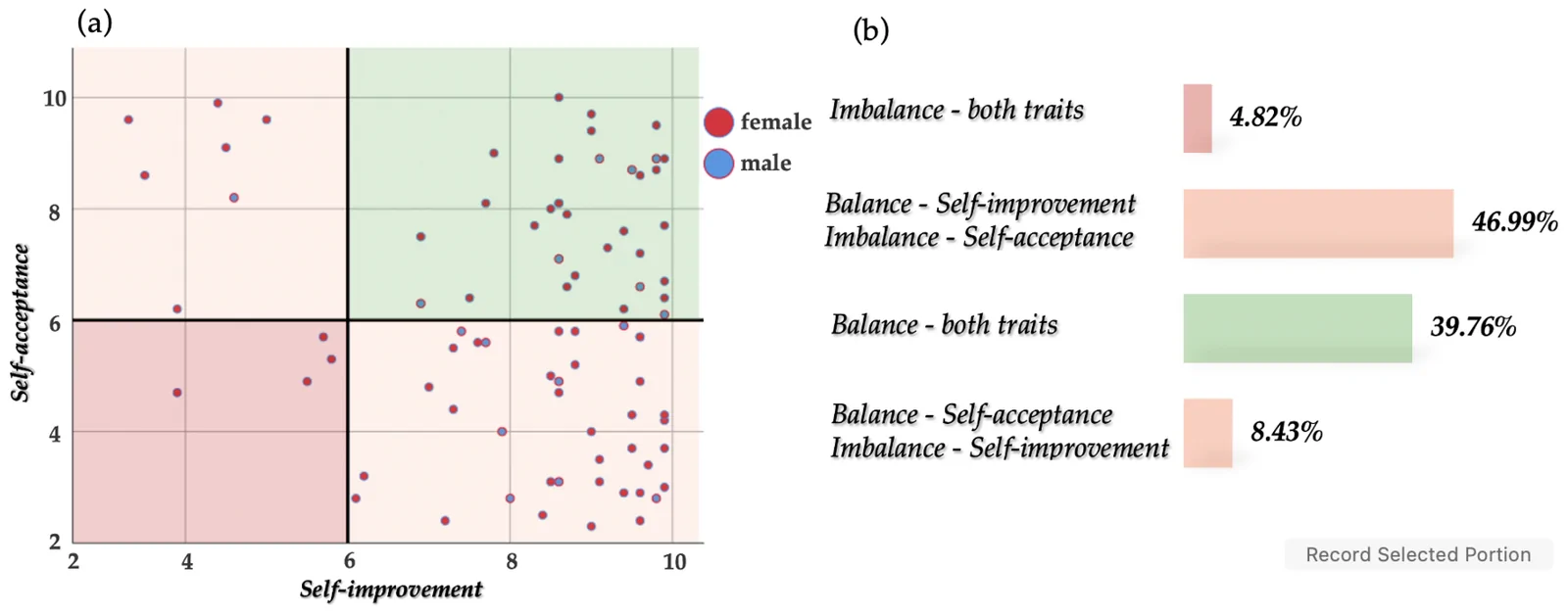
Distribution of participants within the Self-approach trait pair (Self-improvement vs. Self-acceptance): (**a**) by gender and balance quadrants; (**b**) by balance quadrants.

Figure 4a,b show that 8.43% (*n* = 7) of residents were positioned in Quadrant I, corresponding to a self-acceptance-dominant configuration (Self-acceptance ≥ 6, Self-improvement < 6). The largest proportion, 46.99% (*n* = 39), was classified in Quadrant III, reflecting a self-improvement-dominant configuration (Self-improvement ≥ 6, Self-acceptance < 6). 4.82% (*n* = 4) of participants fell within Quadrant IV, indicating that neither tendency reached the neutrality threshold. Finally, 39.76% (*n* = 33) were located in Quadrant II, representing a balanced profile.

Overall, the most frequent configuration in this trait pair involved moderate or higher levels of Self-improvement with comparatively lower levels of Self-acceptance, suggesting that most non-balanced profiles reflected asymmetry between the two complementary tendencies rather than simultaneously low levels in both traits.

#### 3.2.4. The Relationship Control Trait Pair (Assertive–Helpful)

The Relationship control trait pair focuses on the balance between Assertiveness and Helpfulness. In the analyzed group, 39.76% of residents were in the balanced zone, while 60.24% showed a non-balanced configuration. Gender distributions are summarized in Table 6.

Balanced profiles were observed in 40.3% of females and 37.5% of males, with no statistically significant difference (*p* = 1.000).

**Figure 5:**
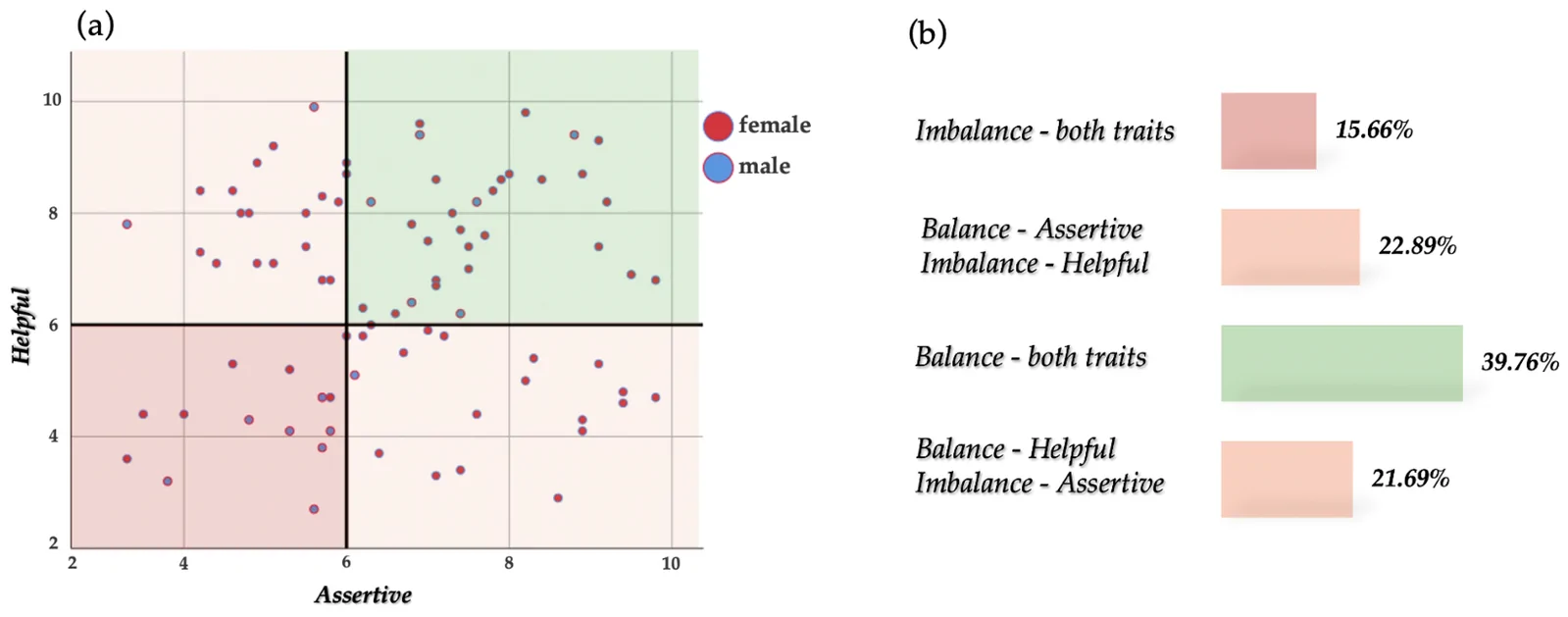
Distribution of participants within the Relationship control trait pair (Assertive vs. Helpful): (**a**) by gender and balance quadrants; (**b**) by balance quadrants.

According to Figure 5a,b, 21.69% (*n* = 18) of participants were positioned in Quadrant I, corresponding to a helpfulness-dominant configuration (Helpful ≥ 6, Assertive < 6). 22.89% (*n* = 19) were classified in Quadrant III, reflecting an assertiveness-dominant configuration (Assertive ≥ 6, Helpful < 6). 15.66% (*n* = 13) of residents fell within Quadrant IV, indicating low levels in both traits. Finally, 39.76% (*n* = 33) were positioned in Quadrant II, representing a balanced relational profile.

These distributions suggest that, for a majority of residents, assertiveness and helpfulness do not reach comparable levels above the neutrality threshold, with most non-balanced profiles reflecting asymmetry between the two complementary tendencies rather than simultaneously low levels in both traits.

#### 3.2.5. The Approach to Opinions Trait Pair (Open/Reflective–Self-Confident)

The Approach to opinions trait pair examines Open/reflective and Self-confident tendencies. At group level, 39.76% of resident physicians showed a balanced configuration and 60.24% showed a non-balanced configuration. The gender comparison yielded a nominal *p* value of 0.049 (Table 7), with balance observed in 62.5% of male and 34.3% of female participants. This was one of six uncorrected comparisons and would not remain significant under a standard multiplicity adjustment; it is therefore reported as an exploratory observation rather than an established gender difference.

**Figure 6:**
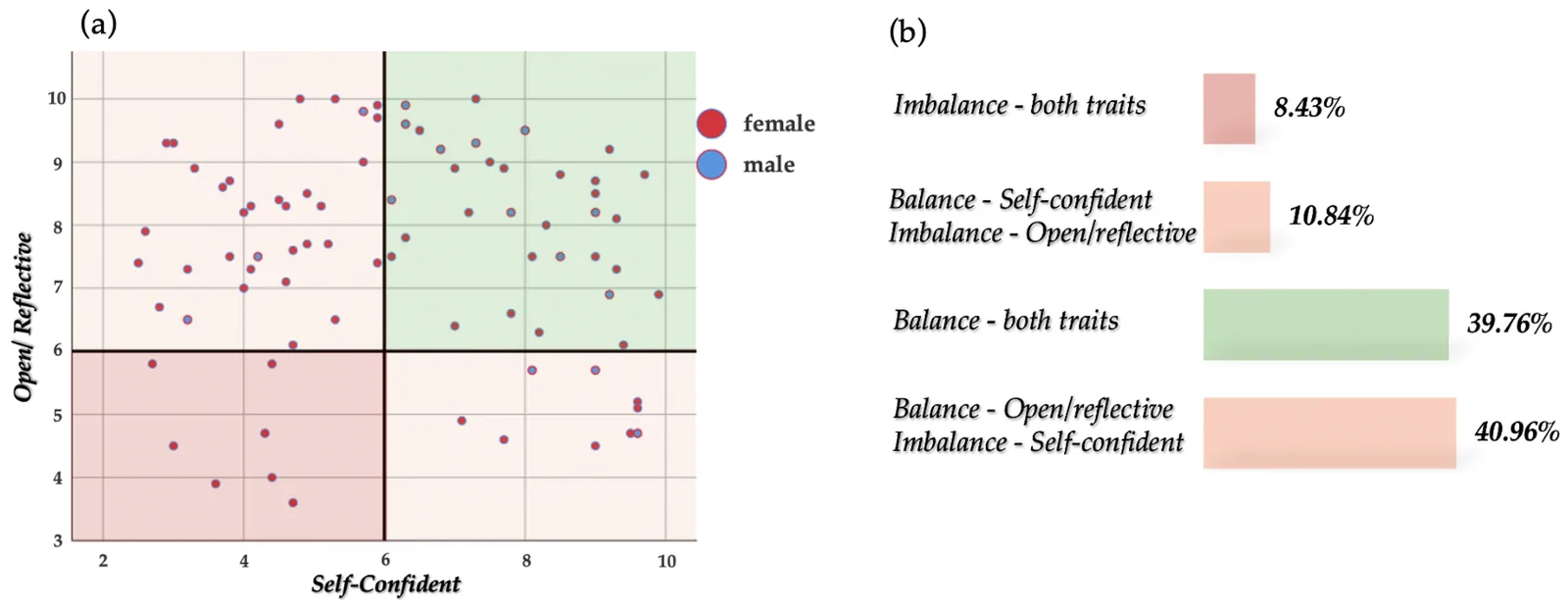
Distribution of participants within the Approach to opinions trait pair (Open/reflective vs. Self-confident): (**a**) by gender and balance quadrants; (**b**) by balance quadrants.

The distribution of residents across quadrants is depicted in Figure 6a,b. 40.96% (*n* = 34) of participants were positioned in Quadrant I, corresponding to an openness-dominant configuration (Open/reflective ≥ 6, Self-confident < 6). 10.84% (*n* = 9) were classified in Quadrant III, reflecting a self-confidence-dominant configuration (Self-confident ≥ 6, Open/reflective < 6). 8.43% (*n* = 7) of residents fell within Quadrant IV, indicating low levels in both traits. Finally, 39.76% (*n* = 33) were located in Quadrant II, representing a balanced profile.

This was the only trait pair in which the gender comparison reached nominal significance. As the six comparisons were not corrected for multiple testing, and as this result would not remain significant under a standard adjustment, it is interpreted guardedly and reported as an exploratory observation rather than as support for a stated hypothesis. It suggests that gender may be associated with the relative configuration of openness and confidence in relation to opinions, while also indicating that most non-balanced profiles reflected asymmetry between the two complementary tendencies rather than simultaneously low levels in both traits.

#### 3.2.6. The Delegation Trait Pair (Assumed Authority–Collaboration)

The Delegation trait pair evaluates the interaction between Assumed authority and Collaboration. This trait pair holds particular significance due to its direct impact on organizational culture. Within the analyzed target group, 55.42% of medical residents fell within the balanced quadrant, while 44.58% showed a non-balanced configuration. As shown in Table 8, balance was more frequently observed among female residents (59.7%) than among male residents (37.5%), although this difference did not reach statistical significance (*p* = 0.161).

**Figure 7:**
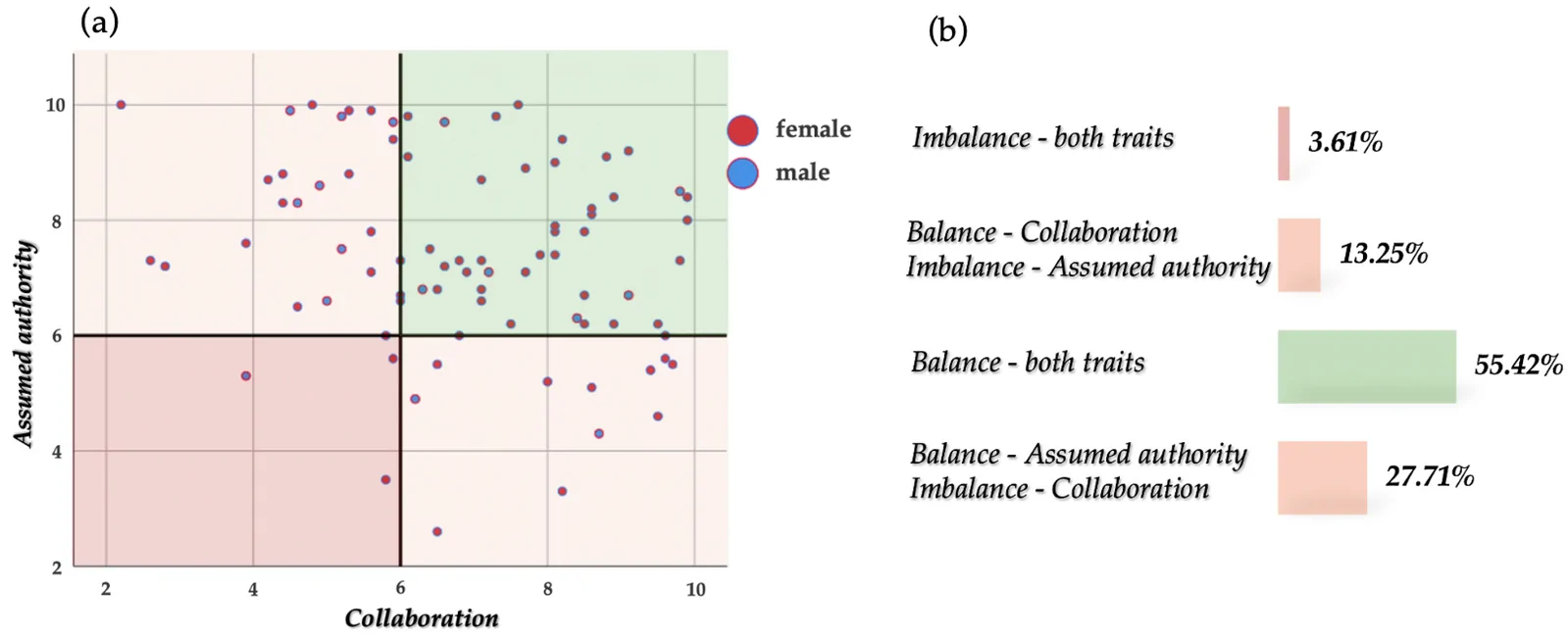
Distribution of participants within the Delegation trait pair (Collaboration vs. Assumed authority): (**a**) by gender and balance quadrants; (**b**) by balance quadrants.

Figure 7a,b show that 27.71% (*n* = 23) of participants were positioned in Quadrant I, corresponding to an authority-dominant configuration (Assumed authority ≥ 6, Collaborative < 6). 13.25% (*n* = 11) were classified in Quadrant III, reflecting a collaboration-dominant configuration (Collaborative ≥ 6, Assumed authority < 6). 3.61% (*n* = 3) of residents fell within Quadrant IV, indicating a configuration in which neither tendency reached the neutrality threshold. Finally, 55.42% (*n* = 46) were located in Quadrant II, representing a balanced decision-making profile.

Among the six trait pairs, Delegation showed the highest proportion of balanced profiles, suggesting that many residents report comparable levels of authority-taking and collaboration, while the non-balanced configurations were predominantly characterized by asymmetry between the two complementary tendencies rather than simultaneously low levels in both traits.

### 3.3. Identified Profiles

The analyses summarized above examined individual traits and their organization into trait pairs. To obtain an integrative view of behavioral configurations, we performed a latent class analysis based on eight binary indicators: the balance status of the six predefined trait pairs together with the two standalone dimensions, Organization and Warmth/empathy. The selected two-class solution showed adequate classification quality (maximum log-likelihood = −403.5756, AIC = 841.1513, BIC = 882.2715, entropy = 0.793). Estimated class population shares were 17.8% and 82.2%, with modal class memberships of 18.1% and 81.9%, respectively. The resulting class distribution is presented in Table 9.

Class 1 comprised 15 residents (18.1%). Estimates based on a class of this size are necessarily imprecise, and the description below is offered as a characterization of the present cohort rather than as a generalizable profile.

According to Table 9, both latent classes were characterized by comparable levels of balance in Communication and Organization. The main differences emerged in Strategic thinking (*p* < 0.001), Self-approach (*p* = 0.022), Relationship control (*p* < 0.001), Approach to opinions (*p* < 0.001), Delegation (*p* = 0.009) and Warmth/Empathy (*p* = 0.002):

- ***Class 1*** (18.1% of residents) showed higher levels of balance in Relationship control (93.3%), Delegation (86.7%) and Warmth/Empathy (100%) and lower balance in Strategic thinking (0%), Self-approach (13.3%) and Approach to opinions (0%).
- ***Class 2*** (81.9% of residents) showed the opposite configuration, with lower balance in Relationship control (27.9%), Delegation (48.5%) and Warmth/Empathy (60.3%), but markedly higher balance in Strategic thinking (47.1%), Self-approach (45.6%) and Approach to opinions (48.5%). No statistically significant differences in latent class membership by gender were identified (*p* = 0.724).

## 4. Discussion

### 4.1. General Patterns of Behavioral Tendencies

#### 4.1.1. Trait-Level Patterns

The study provides a multidimensional description of self-reported behavioral tendencies in this cohort. Most medians were near the instrument’s neutrality threshold, while the observed ranges showed substantial between-participant variation. These data describe the cohort at one time point; they do not show whether tendencies are unconsolidated, predictably activated in practice, or changing with training. Trait-pair analysis adds a configuration-level view of how complementary preferences co-occur.

#### 4.1.2. Configuration-Level Asymmetries

Across five of the six trait pairs, non-balanced configurations represented 60.24–65.06% of participants, and Delegation showed a larger balanced group (55.42%). These are configuration-level differences observed at a single assessment. The cross-sectional design cannot distinguish between-individual variation from with-in-individual change, and no developmental sequence is inferred.

The interpretations that follow are offered as hypotheses about the possible practical significance of the observed configurations. They are drawn from the existing literature rather than from the present data: no outcome, performance or wellbeing measure was collected in this study, and no configuration observed here has been shown to be associated with any clinical consequence.

For *Communication*, the cited literature suggests that pronounced imbalance between directness and diplomacy may be relevant to the delivery of difficult messages and to interpersonal friction [10,28,43,44,45,46,47,48,49,50]. This possible practical significance was not tested here.

For *Strategic thinking*, the prior literature provides reasons to examine whether risk-analysis-dominant and optimism-dominant configurations relate to hesitation, premature closure, or defensive practice [6,15,50,51,52,53]. The present data establish none of these outcomes.

For *Approach to opinions*, openness-dominant and self-confidence-dominant configurations may warrant future study in relation to decisiveness and receptiveness to alternative views [2,5,6,7,9,54]. These labels do not represent measured impairment.

For *Delegation*, authority-dominant and collaboration-dominant configurations may be examined in future research against team-process outcomes [49]; no team outcome was measured in this study.

*Relationship control* and *Self-approach* configurations likewise provide hypotheses for future criterion-validation research concerning assertiveness, helpfulness, self-acceptance, and self-improvement [8,13,45,47,55].

Taken together, the distributions demonstrate heterogeneity in how complementary tendencies co-occur within this cohort. They do not establish routes, stages, or progression toward a preferred endpoint.

#### 4.1.3. Latent Class Profiles as Distinct Behavioral Configurations

Latent class analysis (LCA) distinguished two configuration profiles within this cohort: Class 1—Relational-dominant profile (18.1%) and Class 2—Cognitive-dominant profile (81.9%). The labels describe which domains were more frequently balanced within each class at the time of assessment; they do not imply a developmental sequence, which a single-occasion design cannot establish.

Residents assigned to the Relational-dominant profile showed higher observed balance in Relationship control (93.3%), Delegation (86.7%), and Warmth/Empathy (100%), and lower observed balance in Strategic thinking (0%), Self-approach (13.3%), and Approach to opinions (0%). These percentages describe co-occurrence within 15 participants at one assessment and do not establish that one domain develops before another.

Residents assigned to the Cognitive-dominant profile showed higher observed balance in Strategic thinking (47.1%), Self-approach (45.6%), and Approach to opinions (48.5%) than the smaller class, alongside lower observed balance in Relationship control (27.9%), Delegation (48.5%), and Warmth/Empathy (60.3%). The data cannot determine whether this configuration reflects prior individual differences, training exposure, or change over time. No gender difference in latent class membership was observed (*p* = 0.724).

A comparable person-centered approach has recently been applied in undergraduate medical education, where latent profile analysis of professional identity formation, professionalism, leadership, resilience and well-being distinguished several student sub-groups [56]. The present findings extend that line of work to postgraduate trainees and to complementary behavioral tendencies, while sharing the same interpretive caution: profiles derived cross-sectionally describe subgroups within a cohort rather than stages through which individuals pass.

Neither latent class is interpreted as a developmental stage, a disadvantage, or a population subtype. Both are descriptive summaries of the participating cohort and require replication.

#### 4.1.4. Context for Future Criterion Validation

The following literature-based considerations identify external outcomes that future validation studies could examine; they are not outcomes of the present study:

- At *physician level*, future studies could test whether Strategic thinking, Self-approach, or Delegation configurations are associated with decision timing, wellbeing, or professional functioning. Recent Romanian studies provide relevant national context on depressive symptoms, sleep, physical activity, and mental health among medical trainees [57,58], but these variables were not assessed here and no association is implied.
- At *patient level*, future criterion measures could include communication quality, informed-consent processes, and patient satisfaction [45,46,50].
- At *organizational level*, future studies could examine team coordination, conflict, and decision-making processes in relation to Relationship control and Delegation configurations [50,54].

### 4.2. Potential Educational Implications for Future Testing

No educational method or intervention was evaluated. The following points are therefore framed as questions for future study rather than as recommendations demonstrated by the present data.

**Figure 8:**
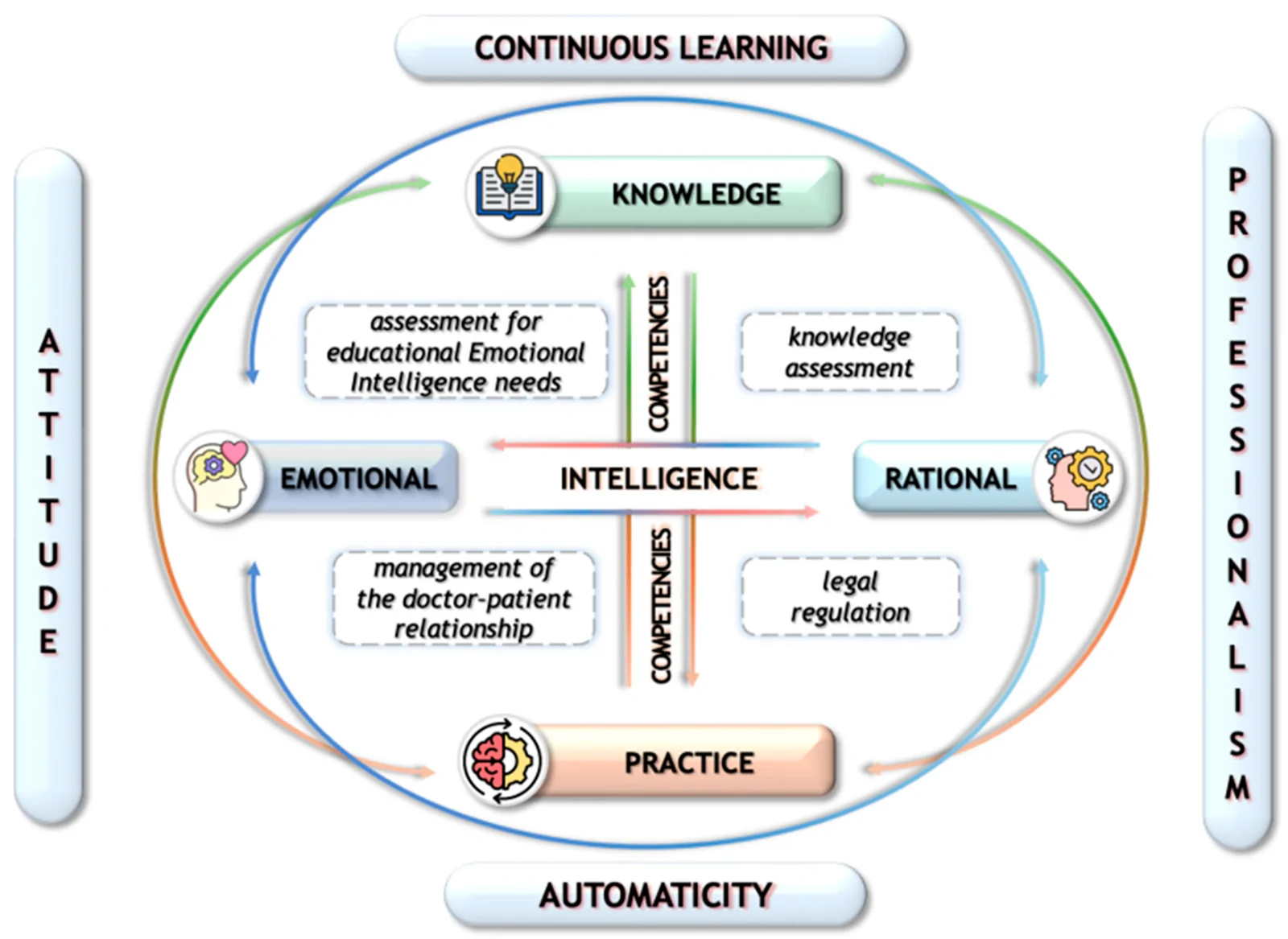
The Physician’s Compass 360°: a conceptual model proposed for future testing, not an empirical output of the present study.

- Future research could test whether structured reflection or self-awareness activities alter complementary tendency configurations or relevant educational outcomes.
- It could also examine whether configuration-level feedback adds value beyond continuous trait scores and established personality measures.
- Finally, the Physician’s Compass 360° is presented as a conceptual model proposed for future testing, not as an empirical output of this study. It was not derived from these data, its four domains were not measured, and no analysis reported here tests the model (Figure 8).

The model groups Knowledge, Clinical Application, Rationality, and Emotional Awareness as conceptual domains. The vertical axis connects Knowledge, or “know-what”, with Clinical Application, or “know-how”, representing the relationship between learning and its translation into practice. The horizontal axis connects Rationality with Emotional Awareness, reflecting the proposed complementarity between analytical judgment and sensitivity to patients’ individual and relational needs. The intersections suggest four areas for future investigation: emotional awareness in relation to educational needs; the integration of knowledge with ethical and rational judgment; the application of ethical and legal principles in clinical practice; and the adaptation of clinical behavior to patients’ emotional and contextual needs.

### 4.3. Strengths

The principal strength is the configuration-level analysis of predefined complementary tendency pairs, which describes asymmetries that isolated trait summaries do not show. The analysis also reports both continuous distributions and four-category configurations, allowing the binary aggregation to be interpreted in context.

### 4.4. Limitations

The study is exploratory, cross-sectional, self-reported, and based on 83 volunteers from one university. Self-selection cannot be quantified because no information was collected from non-participants. The cohort combined General Medicine and Dental Medicine, which differ in licensing pathway and clinical exposure; sparse cells precluded interpretable track-stratified analyses, so comparisons between tracks should not be drawn. No benchmark group was included, and the observed configurations cannot be considered specific to residency. The Harrison Assessment has limited independent peer-reviewed evidence and no identified physician-specific validation; item-level responses were unavailable in the analytical dataset, so internal consistency, construct validity, and gender measurement invariance were not evaluated. Dichotomising scores at the provider-defined threshold discards information, and alternative thresholds were not examined. Six gender comparisons were uncorrected for multiplicity. The LCA used eight binary indicators in a small sample and included a modal class of 15 participants; no resampling, split-sample validation, or independent replication was performed. The latent classes are therefore sample-specific descriptions, not established population subtypes. No clinical, educational, performance, or wellbeing outcome was measured. The data do not support developmental, predictive, diagnostic, or intervention-effect claims, and the Physician’s Compass 360° remains unvalidated. Consistent with this, the Harrison Assessment is treated solely as an exploratory measure of self-reported behavioral tendencies rather than as a validated index of behavioral competence or clinical functioning, and its psychometric adequacy in resident-physician populations remains to be independently established.

### 4.5. Future Research

Building on these findings, future research should investigate the psychometric validity, cross-cultural applicability, and predictive accuracy of the Harrison Assessment by using longitudinal studies. More research is also needed to evaluate whether the instrument can effectively predict real-world workplace behavior under varying organizational, cultural, and high-pressure contexts. Additionally, comparative studies between Harrison Assessment and scientifically acknowledged personality frameworks, such as Big Five or HEXACO model, could provide stronger empirical evidence regarding its reliability and behavioral validity. Specifically, future research should (1) track how trait pair configurations change longitudinally as early-career physicians transition into independent practice; (2) test targeted educational interventions addressing key imbalances (e.g., communication, emotional regulation, clinical reasoning, leadership); and (3) replicate the study across multiple training centers and cultural contexts to strengthen external validity. In addition, psychometric profiles should be linked to objective outcomes (e.g., clinical performance, patient satisfaction, error rates), and the Compass dimensions should be operationalized into measurable variables to enable quantitative validation and predictive testing. Finally, extending the same approach to students may support earlier self-awareness and structured reflection across training stages.

## 5. Conclusions

This exploratory cross-sectional study describes heterogeneity in self-reported behavioral tendencies among 83 resident physicians. For five trait pairs, non-balanced configurations were more frequent than balanced configurations; in Delegation, a balanced configuration was more frequent. The latent class solution summarizes patterns within this cohort but is statistically fragile and requires replication. The findings do not establish competence, clinical consequence, developmental progression, or intervention need. Whether configuration-level profiling or the Physician’s Compass 360° has educational, coaching, or predictive value must be tested in larger multi-center samples using population-validated measures, longitudinal designs, and external outcomes.

## Figures and Tables

**Table 1:** General demographic profile of the target group (n = 83).

*Variable*	*Category*	*n (%)*
Gender	Female	67 (80.7%)
	Male	16 (19.3%)
Graduation field	Dental Medicine	45 (54.2%)
	General Medicine	38 (45.8%)

**Table 2:** Descriptive profile of traits among the resident physicians participating in the study.

*Parameter*	*Description*	*Mean* *± SD*	*Median* *(Inter-Quartile Range)*	*Min.*	*Max.*	*% of Respondents* *Exhibiting Strong* *Innate Trait*
***Diplomatic***	Tendency to express things tactfully, in a skillful and elegant manner	6.16 ± 1.96	6.4 (4.5–7.6)	2.3	10	*10.84%*
***Frank/*** ***direct***	Tendency to communicate openly and straightforwardly, saying what one truly thinks	6.82 ± 1.77	7.2 (5.3–8.3)	2.8	9.9	*10.84%*
***Risk*** ***analyzer***	Tendency to identify potential difficulties or risks before making a decision or taking action	6.84 ± 1.77	7 (5.7–8)	3.4	9.9	*16.87%*
***Optimistic***	Tendency to maintain a positive attitude and to focus on the favorable aspects of situations	6.49 ± 2.27	6.6 (4.5–8.7)	2.7	9.9	*21.69%*
***Self-acceptance***	Tendency to acknowledge and accept one’s own limitations, mistakes, or imperfections	6.04 ± 2.25	5.8 (4.2–8.1)	2.3	10	*10.84%*
***Self-improvement***	Tendency to set ambitious goals and continuously seek personal and professional growth	8.23 ± 1.73	8.7 (7.5–9.6)	3.3	9.9	*44.58%*
***Helpful***	Tendency to offer spontaneous help and support when someone is in need	6.59 ± 1.91	6.8 (4.8–8.2)	2.7	9.9	*8.43%*
***Assertive***	Willingness to express personal needs and desires clearly and confidently	6.65 ± 1.66	6.7 (5.5–7.8)	3.3	9.8	*10.84%*
***Open/*** ***reflective***	Tendency to consider and reflect on multiple, diverse points of view	7.55 ± 1.67	7.7 (6.5–8.9)	3.6	10	*21.69%*
***Self-*** ***confident***	Tendency to trust in one’s own judgments and opinions	6.22 ± 2.22	6.1 (4.4–8.2)	2.5	9.9	*20.48%*
***Assumed*** ***authority***	Desire to take decision-making authority and willingness to assume related responsibilities	7.38 ± 1.69	7.3 (6.2–8.7)	2.6	10	*20.48%*
***Collaborative***	Tendency to cooperate, work effectively with others, and value teamwork	6.92 ± 1.86	6.9 (5.6–8.5)	2.2	9.9	*14.46%*
***Organized***	Tendency to plan, structure, and maintain order in daily activities	7.89 ± 1.48	8.2 (7–9.2)	3.9	9.8	*28.91%*
***Warm/*** ***empathic***	Tendency to express positive feelings and emotional connection toward others	7.17 ± 2.08	7.5 (5.5–9.1)	3	10	*30.12%*

**Table 3:** Distribution of participants according to the Communication trait pair and gender.

*Balance Status*	*Female*		*Male*		p *
	*N*	*%*	*N*	*%*	
***Imbalance***	43	64.2%	11	68.8%	1.000
***Balance***	24	35.8%	5	31.2%	

* Fisher’s Exact Test, Cramér’s V = 0.038.

**Table 4:** Distribution of participants according to the Strategic thinking trait pair and gender.

*Balance Status*	*Female*		*Male*		p *
	*n*	*%*	*n*	*%*	
***Imbalance***	43	64.2%	8	50%	0.393
***Balance***	24	35.8%	8	50%	

* Fisher’s Exact Test, Cramér’s V = 0.115.

**Table 5:** Distribution of participants according to the Self-approach trait pair and gender.

*Balance Status*	*Female*		*Male*		p *
	*n*	*%*	*n*	*%*	
***Imbalance***	41	61.2%	9	56.3%	0.780
***Balance***	26	38.8%	7	43.8%	

* Fisher’s Exact Test, Cramér’s V = 0.040.

**Table 6:** Distribution of medical residents according to the relationship control trait pair and gender.

Balance *Status*	*Female*		*Male*		p *
	*n*	*%*	*n*	*%*	
***Imbalance***	40	59.7%	10	62.5%	1.000
***Balance***	27	40.3%	6	37.5%	

* Fisher’s Exact Test, Cramér’s V = 0.023.

**Table 7:** Distribution of medical residents according to the Approach to opinions trait pair and gender.

*Balance Status*	*Female*		*Male*		*p* *
	*n*	*%*	*n*	*%*	
***Imbalance***	44	65.7%	6	37.5%	**0.049**
***Balance***	23	34.3%	10	62.5%	

* Fisher’s Exact Test, Cramér’s V = 0.227.

**Table 8:** Distribution of medical residents according to the Delegation trait pair and gender.

*Balance Status*	*Female*		*Male*		*p* *
	*n*	*%*	*n*	*%*	
***Imbalance***	27	40.3%	10	62.5%	0.161
***Balance***	40	59.7%	6	37.5%	

* Fisher’s Exact Test, Cramér’s V = 0.176.

**Table 9:** Distribution of resident physicians assigned to the two latent classes identified by latent class analysis, in relation to the analyzed indicators.

*Parameter (Balanced)*	*Class 1 (N, %)*	*Class 2 (N, %)*	*p* *
***Total Number, %***	15 (18.1%)	68 (81.9%)	-
***Communication***	7 (46.7%)	22 (32.4%)	0.372
***Strategic thinking***	0 (0%)	32 (47.1%)	**<0.001**
***Self-approach***	2 (13.3%)	31 (45.6%)	**0.022**
***Relationship control***	14 (93.3%)	19 (27.9%)	**<0.001**
***Approach to opinions***	0 (0%)	33 (48.5%)	**<0.001**
***Delegation***	13 (86.7%)	33 (48.5%)	**0.009**
***Organization***	15 (100%)	59 (86.8%)	0.201
***Warmth/Empathy***	15 (100%)	41 (60.3%)	**0.002**

* Fisher’s Exact Test, Final latent class model: maximum log-likelihood = −403.5756, AIC = 841.1513, BIC = 882.2715, entropy = 0.793. Estimated class population shares: 17.8% and 82.2%; modal class memberships: 18.1% and 81.9%.

## Data Availability

The data presented in this study are available upon request from the corresponding author. The data are not publicly available due to participant privacy and confidentiality considerations.

## References

[1] Katajavuori N., Lindblom-Ylänne S., Hirvonen J. (2006). The significance of practical training in linking theoretical studies with practice. High. Educ..

[2] Van Ryneveld L., Holm D.E., Cronje T., Leask R. (2020). The impact of practical experience on theoretical knowledge at different cognitive levels. J. S. Afr. Vet. Assoc..

[3] Collin K., Tynjälä P. (2003). Integrating theory and practice? Employees’ and students’ experiences of learning at work. J. Workplace Learn..

[4] Fantinelli S., Cortini M., Di Fiore T., Iervese S., Galanti T. (2024). Bridging the gap between theoretical learning and practical application: A qualitative study in the Italian educational context. Educ. Sci..

[5] Montag C., Sindermann C., Lester D., Davis K.L. (2020). Linking individual differences in satisfaction with each of Maslow’s needs to the Big Five personality traits and Panksepp’s primary emotional systems. Heliyon.

[6] De Oliveira D.F., Balbino C.M., Ribeiro C.B., De Oliveira Ramos R.M., Sepp V.J., Loureiro L.H. (2023). Frederick Herzberg and the theory of the two factors in the contribution to the prevention of absenteeism at work. Cuad. Educ. Desarro..

[7] Conger D.S., Mullen D. (1981). Life skills. Int. J. Adv. Couns..

[8] Lynch D. (2017). Self-evaluation: Building student self-awareness and competence. Canadian Engineering Education Association (CEEA17) Conference.

[9] Williams G.W., Nel B.P. (2023). Teachers’ professional development shaped through self-reflection using video-stimulated recall. Afr. J. Teach. Educ. Dev..

[10] Goleman D. (2005). Emotional Intelligence.

[11] Saeedi N., Pazvari M., Masouleh S., Mousavian S. (2012). Studying the influence of emotional intelligence on career success. J. Basic. Appl. Sci. Res..

[12] Antonopoulou H. (2024). The value of emotional intelligence: Self-awareness, self-regulation, motivation, and empathy as key components. Tech. Educ. Humanit..

[13] Gayatri B. (2022). Managing emotional intelligence: A key element for success. Int. J. Sci. Res. Publ..

[14] Bennett N., Lemoine G.J. (2014). What a difference a word makes: Understanding threats to performance in a VUCA world. Bus. Horiz..

[15] Murugan S., Rajavel S., Aggarwal A., Singh A. (2020). Volatility, uncertainty, complexity and ambiguity (VUCA) in the con-text of the COVID-19 pandemic: Challenges and way forward. Int. J. Health Syst. Implement. Res..

[16] Horney N., Pasmore B., O’Shea T. (2010). Leadership agility: A business imperative for a VUCA world. People Strategy.

[17] Andersson J., De Paula T. (2025). Events in VUCA Environments: Coping with the COVID-19 Pandemic in the Swedish Event Industry. Master’s Thesis.

[18] Peña A. (2010). The Dreyfus model of clinical problem-solving skills acquisition: A critical perspective. Med. Educ. Online.

[19] Dreyfus S.E. (2004). The Five-Stage model of adult skill acquisition. Bull. Sci. Technol. Soc..

[20] Uchino R., Yanagawa F., Weigand B., Orlando J.P., Tachovsky T.J., Dave K.A., Stawicki S.P. (2015). Focus on emotional intelligence in medical education: From problem awareness to system-based solutions. Int. J. Acad. Med..

[21] Dewsnap M.A., Arroliga A.C., Adair-White B.A. (2021). The lived experience of medical training and emotional intelligence. Bayl. Univ. Med. Cent. Proc..

[22] Sulistio M.S., Khera A., Squiers K., Sanghavi M., Ayers C.R., Weng W., Kazi S., De Lemos J., Johnson D.H., Kirk L. (2019). Effects of gender in resident evaluations and certifying examination pass rates. BMC Med. Educ..

[23] Graf J., Smolka R., Simoes E., Zipfel S., Junne F., Holderried F., Wosnik A., Doherty A.M., Menzel K., Herrmann-Werner A. (2017). Communication skills of medical students during the OSCE: Gender-specific differences in a longitudinal trend study. BMC Med. Educ..

[24] Cernega A., Nicolescu D., Meleșcanu Imre M., Ripszky Totan A., Arsene A., Șerban R., Perpelea A.-C., Nedea M.-I., Pițuru S.-M. (2024). Volatility, uncertainty, complexity and ambiguity (VUCA) in healthcare. Healthcare.

[25] Woo S.E., Hofmans J., Wille B., Tay L. (2023). Person-Centered Modeling: Techniques for Studying Associations Between People Rather than Variables. Annu. Rev. Organ. Psychol. Organ. Behav..

[26] Peroš A., Bralić N., Buljan I. (2025). Personality traits and medical specialty preference among medical students and graduates: A scoping review. Croat. Med. J..

[27] Howard M.C., Hoffman M.E. (2017). Variable-Centered, Person-Centered, and Person-Specific approaches. Organ. Res. Methods.

[28] Pflügner K., Maier C., Mattke J., Weitzel T. (2020). Personality Profiles that Put Users at Risk of Perceiving Technostress. Bus. Inf. Syst. Eng..

[29] Soto C.J., John O.P. (2017). The next Big Five Inventory (BFI-2): Developing and assessing a hierarchical model with 15 facets to enhance bandwidth, fidelity, and predictive power. J. Personal. Soc. Psychol..

[30] Pittenger D.J. (1993). The Utility of the Myers-Briggs Type Indicator. Rev. Educ. Res..

[31] Ashton M.C., Lee K. (2020). Objections to the HEXACO Model of Personality Structure—And Why Those Objections Fail. Eur. J. Pers..

[32] Sellbom M. (2025). MMPI-3 Assessment of Externalizing Psychopathology in Targeted Community and University Samples. Assessment.

[33] Soto C.J. (2019). How Replicable Are Links Between Personality Traits and Consequential Life Outcomes? The Life Outcomes of Personality Replication Project. Psychol. Sci..

[34] Laajaj R., Macours K., Pinzon Hernandez D.A., Arias O., Gosling S.D., Potter J., Rubio-Codina M., Vakis R. (2019). Challenges to capture the big five personality traits in non-WEIRD populations. Sci. Adv..

[35] Alnafisah A. (2026). Personality, capability, and context: A 30 year integrative review and theoretical expansion of the Big Five framework. Front. Psychol..

[36] Fleeson W., Jayawickreme E. (2015). Whole Trait Theory. J. Res. Pers..

[37] Bleidorn W., Hill P.L., Back M.D., Denissen J.J.A., Hennecke M., Hopwood C.J., Jokela M., Kandler C., Lucas R.E., Luhmann M. (2019). The policy relevance of personality traits. Am. Psychol..

[38] Harrison Assessments. https://www.harrisonassessments.com/.

[39] Craigen B. (2013). The Relationship Between Personality Traits, Interests, and Job Performance (Doctoral dissertation). ProQuest Dissertations & Theses Global.

[40] Gajula S., Chintalapuri B. (2016). To Arrive At Benchmarking For Through Evaluation of Job Performance and Be-havioural Assessment. Int. J. Indian Psychol..

[41] Dawson Consulting Group (2020). Understanding Harrison Assessments. https://www.dawsonconsultinggroup.com/fexqpjld0m9/wp-content/uploads/2020/02/HA-Career-Navigator-Sample-Career-Development-v2.pdf.

[42] Trusted Coach (2015). Reliability and Validity of Harrison Assessments. https://www.trustedcoach.com/wp-content/uploads/2015/01/HATS_Reliability_and_Validity.pdf.

[43] Shoss M.K., Witt L.A. (2015). Trait Interactions and Other Configural Approaches to Personality. Handbook of Personality at Work.

[44] Harvard Division of Continuing Education (2024). Emotional Intelligence is no Soft Skill. https://professional.dce.harvard.edu/blog/emotional-intelligence-is-no-soft-skill/.

[45] Hickson G.B. (1994). Obstetricians’ prior malpractice experience and patients’ satisfaction with care. JAMA.

[46] Clayton E.W., Hickson G.B., Pichert J., Federspiel C. (1997). Development of an early identification and response model of malpractice prevention. Law Contemp. Probl..

[47] Reichel A., Schiffinger M., Chudzikowski K., Demel B., Mayrhofer W., Schneidhofer T., Steyrer J. Organizing career success: An exploratory study of individuals’ configuration of objective and subjective career success. Proceedings of the 22nd EGOS Colloquium.

[48] Hickson G.B., Clayton E.W., Githens P.B., Sloan F.A. (1992). Factors that prompted families to file medical malpractice claims following perinatal injuries. JAMA.

[49] Garattini L., Padula A. (2019). Defensive medicine in Europe: A “full circle”?. Eur. J. Health Econ..

[50] Bester J.C. (2020). Defensive practice is indefensible: How defensive medicine runs counter to the ethical and professional obligations of clinicians. Med. Health Care Philos..

[51] Studdert D.M., Mello M.M., Sage W.M., DesRoches C.M., Peugh J., Zapert K., Brennan T.A. (2005). Defensive medicine among high-risk specialist physicians in a volatile malpractice environment. JAMA.

[52] Cernega A., Meleșcanu Imre M., Ripszky Totan A., Arsene A., Dimitriu B., Radoi D., Ilie M.-I., Pițuru S.-M. (2023). Collateral victims of defensive medical practice. Healthcare.

[53] Hvidt E.A., Lykkegaard J., Pedersen L.B., Pedersen K.M., Munck A., Andersen M.K. (2017). How is defensive medicine un-derstood and experienced in a primary care setting? A qualitative focus group study among Danish general practitioners. BMJ Open.

[54] Sanchez-García M.F., Suárez-Ortega M. (2021). Professional success and satisfaction in career development. Rev. Investig. Educ..

[55] Hildred K., Piteira M., Cervai S., Pinto J.C. (2023). Objective and subjective career success: Individual, structural, and behavioral determinants in European hybrid workers. Front. Psychol..

[56] Nsimbe D., Hickey A., Harkin D.W., Bensaaud A., Ryan Á., Doyle F. (2026). Latent Profiles of Professional Identity Formation, Profes-sionalism, Leadership, Resilience, and Well-being among Medical Students. Med. Sci. Educ..

[57] Duica C.L., Negoita S.I., Pleșea-Condratovici A., Moroianu L.-A., Ignat M.D., Nicolcescu P., Ciubara A., Robles-Rivera K., Mititelu-Tartau L., Pleșea-Condratovici C. (2025). Depression in Romanian Medical Students: A Study, Systematic Review, and Me-ta-Analysis. J. Clin. Med..

[58] Pleșea-Condratovici C., Pleșea-Condratovici A., Negoita S.I., Stoian V.-I., Moroianu L.-A., Baroiu L. (2025). Sleep Quality and Sex-Specific Physical Activity Benefits Predict Mental Health in Romanian Medical Students: A Cross-Sectional Analysis. J. Clin. Med..

